# IL-25 Inhibits Atherosclerosis Development in Apolipoprotein E Deficient Mice

**DOI:** 10.1371/journal.pone.0117255

**Published:** 2015-01-28

**Authors:** Polyxeni T. Mantani, Pontus Dunér, Eva Bengtsson, Ragnar Alm, Irena Ljungcrantz, Ingrid Söderberg, Lena Sundius, Fong To, Jan Nilsson, Harry Björkbacka, Gunilla Nordin Fredrikson

**Affiliations:** 1 Department of Clinical Sciences, Skåne University Hospital Malmö, Lund University, Malmö, Sweden; 2 Faculty of Health and Society, Malmö University, Malmö, Sweden; Baker IDI Heart and Diabetes Institute, AUSTRALIA

## Abstract

**Objective:**

IL-25 has been implicated in the initiation of type 2 immunity and in the protection against autoimmune inflammatory diseases. Recent studies have identified the novel innate lymphoid type 2 cells (ILC2s) as an IL-25 target cell population. The purpose of this study was to evaluate if IL-25 has any influence on atherosclerosis development in mice.

**Methods and Results:**

Administration of 1 μg IL-25 per day for one week to atherosclerosis-prone apolipoprotein (apo)E deficient mice, had limited effect on the frequency of T cell populations, but resulted in a large expansion of ILC2s in the spleen. The expansion was accompanied by increased levels of anti-phosphorylcholine (PC) natural IgM antibodies in plasma and elevated levels of IL-5 in plasma and spleen. Transfer of ILC2s to apoE deficient mice elevated the natural antibody-producing B1a cell population in the spleen. Treatment of apoE/Rag-1 deficient mice with IL-25 was also associated with extensive expansion of splenic ILC2s and increased plasma IL-5, suggesting ILC2s to be the source of IL-5. Administration of IL-25 in IL-5 deficient mice resulted in an expanded ILC2 population, but did not stimulate generation of anti-PC IgM, indicating that IL-5 is not required for ILC2 expansion but for the downstream production of natural antibodies. Additionally, administration of 1 μg IL-25 per day for 4 weeks in apoE deficient mice reduced atherosclerosis in the aorta both during initiation and progression of the disease.

**Conclusions:**

The present findings demonstrate that IL-25 has a protective role in atherosclerosis mediated by innate responses, including ILC2 expansion, increased IL-5 secretion, B1a expansion and natural anti-PC IgM generation, rather than adaptive Th2 responses.

## Introduction

IL-25 (also known as IL-17E), a member of the IL-17 cytokine family, has been implicated in the initiation of type 2 immunity by driving the expression of IL-4, IL-5 and IL-13 [1]. Studies using IL-25 deficient mice have shown that IL-25 influences the Th1/Th17 cell responses. IL-25 deficient mice, when infected with Trichuris muris, develop a severe intestinal inflammation and increased levels of the pro-inflammatory cytokines IL-17A and IFN-γ [2]. In addition, IL-25 deficiency has been shown to induce more severe experimental autoimmune encephalomyelitis, accelerated by increased numbers of inflammatory IL-17 and IFN-γ producing T cells [3]. Taken together, it suggests that IL-25 inhibits development of Th1 and Th17 cells by inducing elevated levels of Th2 cytokines. Furthermore, NOD mice treated with IL-25 demonstrated a diminished frequency of autoreactive Th17 cells per-islet infiltrate but an increase in the T regulatory cell population [4].

Recently, studies of the two type-2 inducing cytokines, IL-25 and IL-33, have identified a novel innate target cell population [5]. The name “innate lymphoid type 2 cells” (ILC2s) has been proposed to be used to cover this cell population [6], previously called innate helper type 2 cells [7], nuocytes [8] or natural helper cells [9]. ILC2s are functionally similar to CD4^+^ Th2 cells [7], but are also more widely distributed in tissues independent of antigenic stimulation [10]. Still innate lymphoid cells have been shown to express MHC class II molecules, indicating that they can present antigens and may also contribute to initiation of T cell responses [8]. In addition, ILC2s have been shown to release IL-5 and IL-13, representing an early source of these cytokines in type-2 immunity [6,8]. In accordance, ILC2s have been attributed important protective functions against parasitic worm infections [5,6]. Recently a study demonstrated the presence of natural helper cells in aortic samples from mice and isolated aortic natural helper cells were found to produce IL-5 in response to IL-33 treatment [11].

B2 cells respond to T cell-dependent antigens, whereas B1 cells seem to be involved mainly in T cell-independent immune responses [12]. B1 cells are the major B cell population in the peritoneal and pleural cavities in mice and the main producers of natural antibodies [12]. These antibodies are specific for self-antigens such as the phosphocholine headgroup of oxidized phospholipids expressed on oxidized low density lipoprotein (LDL) and apoptotic cells [13]. B1 cells expressing CD5 are called B1a cells, whereas a minor subset of B cells that do not express CD5 but closely resemble these CD5^+^ B1a cells are known as B1b cells [12]. Previous experimental findings have shown that conventional B2 cells contribute to atherosclerosis development, whereas peritoneal B1a cells are athero-protective by producing natural IgM [14,15].

Several lines of evidence indicate that adaptive immune responses contribute to the development of atherosclerosis by promoting inflammation and plaque growth [16,17]. However, immunization of hypercholesterolemic animals with native or oxidized LDL unexpectedly resulted in a significant reduction of atherosclerosis development, suggesting that both atherogenic and protective immune responses exist [18,19]. Th1 effector cells are believed to drive the disease, since deletion of Th1 promoting cytokines and transcription factors have been found to reduce the development of atherosclerosis [16,20,21], whereas studies on T regulatory cells have pointed to a protective role [22,23]. Studies of the role of Th2 immune responses in atherosclerosis have given an inconsistent picture. IL-4 has been found to exert both pro- and anti-atherogenic effects depending on the experimental conditions [16,24], whereas IL-5 has been attributed athero-protective properties by inducing natural IgM antibodies specific to epitopes of oxidized LDL [25]. In addition, IL-33 has been suggested to play a protective role in the development of atherosclerosis via the induction of IL-5 and ox-LDL antibodies [26].

In this study we asked, whether administration of IL-25 to apoE deficient mice has any influence on atherosclerosis development. We found an expansion of ILC2s in the spleen, increased levels of IL-5, elevated levels of plasma anti-phosphorylcholine (PC) natural IgM antibodies accompanied with reduced plaque area in the aorta of IL-25 treated mice, indicating that IL-25 protects against atherosclerosis development.

## Materials and Methods

### Mice

**Figure 1:**
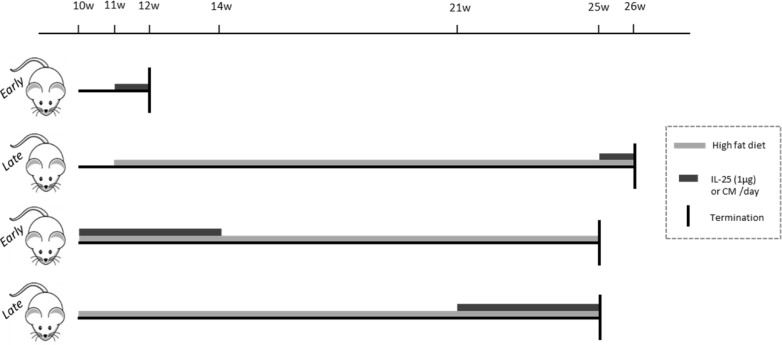
Experimental design of the study. In the one-week treatments of 11 or 25 weeks old *Apoe* ^*-/-*^ mice, the mice were daily injected subcutaneously with 1μg rmIL-25 or control medium (CM = 4 mmol/L HCl). The mice were killed at 12 or 26 weeks of age. In 4-weeks treated mice during 10–14 or 21–25 weeks of age, osmotic pumps containing rmIL-25 or control medium were placed subcutaneously in *Apoe* ^*-/-*^ mice, delivering 1μg of protein/day or equal volume of the control medium. The mice were fed a high fat diet from 10 weeks of age until sacrifice at 25 weeks of age. Grey boxes show the time period of the high fat diet feeding, dark grey boxes the duration of the treatment with IL-25 or control medium and vertical lines the termination point of each experiment.

This study was carried out in strict accordance with the recommendations in the Guide for the Care and Use of Laboratory Animals of the National Institutes of Health. The Local Animal Care and Use Committee at Lund University approved the experimental protocol used in the study (Permit numbers: M153–10, M80–11 and M312–12). All surgery was performed under anesthesia, and all efforts were made to minimize suffering. Osmotic pumps (model 1004, ALZET, DURECT Corporation, Cupertino, CA) containing recombinant mouse IL-25 (rmIL-25, R&D Systems, Abingdon, UK) were surgically placed subcutaneously in either 9–10 or 21 weeks old apoE deficient (*Apoe* ^*-/-*^) mice on C57BL/6 background (Jackson Laboratories, Stock number 002052). The different treatment outlines used in the study are depicted in Fig. 1. The concentration of the solution placed per pump was estimated in order for 1μg of protein to be delivered daily for 4 weeks. For the young mice, after that time period the pumps were surgically removed. A control group of mice where treated with pumps containing control medium (4 mmol/L HCl), the resuspension solution of rmIL-25, which were surgically placed in 9–10 or 21 weeks old mice as described above in order to deliver equal volume of the solution. At the age of 10 weeks all mice were put on high fat diet (HFD; 0.15% cholesterol and 21% fat (Lantmännen, Sweden)) for 15 weeks after which they were killed by i.p. injection of ketamine and xylazine. Upon termination the mice were weighed, blood samples were taken, spleens, aortas and hearts were dissected. Briefly, spleens were harvested and stored in RPMI-1640 (Gibco, Stockholm, Sweden) on ice for further analyses such as flow cytometry, measurements of released cytokine levels and proliferation assays. Blood was collected by cardiac puncture; blood cells were stained with fluorochrome conjugated antibodies for flow cytometric analysis and plasma samples were stored at -80°C until analysis. Mice were whole body perfused with phosphate buffer saline (PBS). Aortas (including aortic arch) were dissected, mounted *en face* and stored in Histochoice (Amresco, Solon, OH, USA). Hearts were dissected and placed in Histochoice or frozen at -80°C. In control experiments, 11 weeks old *Apoe* ^*-/-*^ mice (Jackson) and 13–15 weeks old *IL-5* ^*-/-*^ on C57BL/6 background (Jackson Laboratories, Stock number 003175) were daily injected subcutaneously with 1μg rmIL-25 or control medium for 7 days and killed the day after, whereas 18–20 weeks old *Apoe* ^*-/-*^ */Rag1* ^*-/-*^ (*Apoe* ^*-/-*^ and *Rag1* ^*-/-*^ on C57BL/6 background, both from Jackson were crossed in-house) were given 3 injections during 7 days and killed the day after. Furthermore, 11 weeks old *Apoe* ^*-/-*^ mice (Jackson) were daily injected subcutaneously with 1μg IL-5 (rmIL-5, R&D Systems, Abingdon, UK) or control medium for 7 days and killed the day after. Additionally, 11 weeks old *Apoe* ^*-/-*^ mice (Jackson) were put on HFD for 15 weeks and during the last week treated with IL-25 according to the one-week pattern described above. The number of mice included in each experiment is shown in the figures. All assessments of outcomes were performed blinded.

### Flow cytometry

Splenocytes in single-cell suspension were prepared by pressing each spleen through a 70-μm cell strainer (BD Falcon, Franklin Lakes, NJ, USA). Erythrocytes were removed using red blood cell lysing buffer (Sigma, St. Louis, MO, USA). Cells were cultured in culture medium (RPMI 1640 medium containing 10% heat-inactivated foetal calf serum (FCS), 1 mmol/L sodium pyruvate, 10 mmol/L Hepes, 50 U penicillin, 50 μg/mL streptomycin, 0.05 mmol/L β-mercaptoethanol and 2 mmol/L L-glutamine; GIBCO, Paisley, UK). Blood and spleen cells were stained with fluorochrome conjugated antibodies. All of the antibodies were purchased from Biolegend (San Diego, CA) unless indicated otherwise; CD3-PE/Cy5, CD3-PE/Cy7, CD4-PB, CD25-APC, Foxp3-PE, IL-5-PE, IL-5-APC, IFNγ-PE, IFNγ-FITC, IL-17-APC, ICOS-PB, CD45-APC/Cy7, IL-7ra-FITC, IL-17RB-APC (R&D Systems, Abingdon, UK), IgM-FITC (eBioscience, San Diego, CA), CD5-PE, B220-PE/Cy7, CD11b-PB, IgD-APC, CD19-AF700. Briefly, cells were first incubated with Fc-receptor blocking antibody (FcR; CD16/32, Biolegend, San Diego, CA) for 5 min and then stained with extracellular antibodies for 30 min on ice. In the case of blood cells, erythrocytes were lysed with lysing buffer (BD Pharm Lyse, BD Biosciences, Stockholm, Sweden). After removing the unbound extracellular antibodies by washing with cold PBS containing 1% heat inactivated FCS and 0.5 mmol/L EDTA the cells were resuspended in Fix/Perm solution (eBioscience, San Diego, CA) and incubated for 30 min on ice. Afterwards, cells were washed with permeabilization buffer (eBioscience, San Diego, CA) and incubated with Fc-receptor blocking antibody prior to staining with intracellular antibodies for 30 min at 4°C. The cells that were stained with IL-5 and IFN-γ fluorochrome conjugated antibodies were stimulated with 20 ng/mL PMA, 1 μg/mL Ionomycin and 5 μg/mL Brefeldin A (Sigma-Aldrich, Stockholm, Sweden) for 4h prior to staining while cells that were stained with IL-17A antibodies were stimulated for 18–24h. The cells were washed with permeabilization buffer (e-Bioscience, San Diego, CA) and resuspended in PBS containing 1% heat inactivated FCS and 0.5 mmol/L EDTA and analyzed with a CyAn ADP flow cytometer (Beckman Coulter, High Wycombe, UK). All flow cytometric data were analyzed with FlowJo software (Tree Star, Inc. Ashland, OR). For ILC2s, splenocytes were used in order to immunomagnetically isolate the cells of interest by negative selection with the use of a custom made kit from Stem Cell Technologies Inc. (Vancouver, Canada). The kit contained a cocktail of biotinylated antibodies targeting the following lineage (Lin) markers CD3, CD4, CD8, CD11b, CD11c, CD45R, CD19, Gr-1, FcεRI, NK 1.1 and Ter-119. After isolation according to the company’s instructions the cells of interest were stained with fluorochrome conjugated antibodies such as Lin-Streptavidin PE/Cy7, ICOS-PB, CD45-APC/Cy7, IL-7ra-FITC, IL-17RB-APC and analyzed with the use of flow cytometry. This gating strategy was in accordance with the work presented by Neill et al. [8]. In some cases, following enrichment with the custom made kit from Stem cell Technologies and staining with the fluorochrome conjugated antibodies mentioned above, ILC2s were sorted with FACSAria (BD Biosciences) as Lin^-^CD45^+^IL17RB^+^ICOS^+^IL7ra ^intermediate^ and a simultaneous sorting of Lin^+^CD45^+^IL17RB^+^ cells (S1 Fig.). After sorting, ILC2s were cultured *in vitro* in the presence of IL-7 (10 ng/mL) and IL-33 (10 ng/mL) for 7 days, replenishing the cell culture media every other day. Thereafter the cells were counted and characterized with flow cytometry. In additional experiments FACS sorted and *in vitro* expanded ILC2s (7 days) were stimulated with PMA (20 ng/mL) and Ionomycin (1 μg/mL) for 4h and stained with IL-5-PE, Lin-Streptavidin PE/Cy7, ICOS-PB, CD45-APC/Cy7, IL7ra-FITC, IL17RB-APC and analyzed with the use of flow cytometry.

### ILC2 transfer to *Apoe* ^*-/-*^ mice

*Apoe* ^*-/-*^ mice were injected daily with 1μg of rmIL-25 for 7 days. Thereafter the mice were killed, spleens were dissected, ILC2s were enriched with the custom made kit from Stem Cell Technologies Inc., FACS sorted as Lin^-^CD45^+^IL17RB^+^ICOS^+^IL7ra^intermediate^ and expanded *in vitro* for 9 days in the presence of IL-7 (10ng/mL) and IL-33 (10ng/mL). Next, ILC2s were counted and 0.5 x 10^6^ cells (resuspended in PBS) were transferred i.p. to *Apoe* ^*-/-*^ mice (day 0). On day 3 the mice were euthanized, blood was obtained with cardiac puncture and spleens were dissected. Splenocytes were analyzed with flow cytometry for the presence of B1a cells. In additional experiments, immunomagnetically enriched, FACS sorted and *in vitro* expanded ILC2s (as described above) were stained with Cell Trace Violet (Invitrogen) according to the company’s instructions. Next, 0.5x10^6^ Violet stained ILC2s (or equal volume of PBS) were transferred i.p. to *Apoe* ^*-/-*^ mice (day 0). On day 3, the mice were euthanized, peritoneal lavage was performed and the spleens were dissected. Peritoneal cells and splenocytes were immunomagnetically enriched in ILC2s and analyzed with the use of flow cytometry for possible detection of Violet^+^ cells.

### Cytokine analysis

Splenocytes (1 x 10^6^ cells/well) were cultured in the presence and absence of 1 μg/mL Ionomycin and 20 ng/mL PMA for 24h. Cell culture supernatants were frozen at -80°C until further analysis. Cytokine release by splenocytes and plasma cytokine levels were assessed using Luminex xMAP technology according to the company’s instructions (IFN-γ, IL-2, IL-4, IL-5, IL-6, IL-9, IL-10, IL-12(p40), IL-13, IL-17A). For *in vitro* experiments, splenocytes (1 x 10^6^ cells/well) isolated from both young and old mice treated for one week with control medium (4 mmol/L HCl) were cultured with 50 ng/mL of rmIL-25 or vehicle (4 mmol/L HCl containing 0.1% BSA) for 24h and further stimulated with 1 μg/mL Ionomycin and 20 ng/mL PMA for 24h. For a second set of *in vitro* experiments, ILC2s or Lin^+^CD45^+^IL17RB^+^ cells (1 x 10^6^ cells/ml) were stimulated with PMA (20 ng/mL) and Ionomycin (1 μg/mL) for 24h. Supernatants were collected and stored at -80°C until analysis of the cytokine levels (IL-4, IL-5, IL-6, IL-9, IL-10, IL-13, GM-CSF) with Luminex xMAP technology as described above.

### Splenocyte proliferation

Splenocytes (2 x 10^5^ cells/well) were cultured in 96-well round-bottom plates (Sarstedt, Nümbrecht, Germany) in the presence and absence of 5 μg/mL Con A (Sigma-Aldrich, Stockholm, Sweden) for 72h. To measure DNA synthesis, the cells were pulsed with 1 μCi [methyl-^3^H] thymidine (Amersham, Piscataway, NJ, USA) during the last 20h of culture. Next, the cells were harvested on glass fiber filters using a Filter Mate harvester (Perkin Elmer, Buckinghamshire, UK) and analyzed using a liquid scintillation beta-counter.

### Plaque area staining

*En face* preparations of the descending aortas and aortic arch were dipped in 78% methanol and stained for 40 min in 0.16% Oil Red O dissolved in 78% methanol containing 0.22 mol/L NaOH. The stained plaque areas were quantified using Image pro plus 4.5 software (Media Cybernetics, Bethesda, MD, USA).

### Immunohistochemistry

Hearts were embedded in OCT (Optimal Cutting Temperature; Tissue-Tek, Zoeterwoulde, The Netherlands) and sections of 10 μm thickness were collected. For macrophage and T cell content, subvalvular plaques were stained with MOMA-2 antibody (BMA Biomedicals, Switzerland) and anti-CD3 (Dako A0452) respectively, using rabbit immunoglobulins (Dako, Solna, Sweden) as negative controls. DAB detection kit was used for color development (Vector Laboratories, CA) and the sections were counterstained in haematoxylin. For assessment of the collagen content of the plaques, subvalvular sections were stained with Van Gieson Solution Acid Fuchsin (Sigma-Aldrich). To assess the necrotic core, sections were stained with haematoxylin/eosin and the area was determined as the acellular area, lacking nuclei and cytoplasm, under the fibrous cap of lesions. All stainings were quantified with Image-Pro-Plus 4.5 software (Media Cybernetics).

### Immunoglobulins in plasma

Plasma IgA, IgG1, IgG2a (not expressed in C57BL/6 mice), IgG2b, IgG3 and IgM levels were assessed with the use of Mouse Isotyping Panel 1 Assay kit from Mesoscale according to the company’s instructions (MSD Multi-spot Assays, Mesoscale Discovery, Maryland, USA). Plasma IgE levels were determined with the use of mouse IgE ELISA Kit from Bethyl Laboratories (Bethyl Laboratories Inc, Montgomery, Texas, USA) while IgM antibodies targeting phosphorylcholine were assessed with an ELISA kit from Athera Biotechnologies (Athera Biotechnologies AB, Solna, Sweden) according to the company’s instructions, with the exception of the use of peroxidase-conjugated anti mouse IgM (Jackson ImmunoResearch).

### Statistics

Analysis of data was performed using unpaired *t* test for normally distributed or log transformed skewed data or Mann Whitney test to assess non-normally distributed variables. Data are presented as mean±standard deviation. Analysis was performed using GraphPad Prism 5.01 (Graphpad Software, La Jolla, CA, USA) and a level of P<0.05 was considered significant.

## Results

### Rationale for the IL-25 treatment

To evaluate the role of IL-25 in the progress of atherosclerosis, atherosclerosis-prone apoE deficient mice at different ages were treated with IL-25 for 4 weeks, both during early and late atherosclerosis development. Due to ethical reasons, daily injections of the cytokine for a long time period were avoided. Accordingly, a 4-week osmotic pump delivering 1 μg IL-25 per day or control medium was used. To examine if IL-25 has any acute influence on immune cell populations and/or cytokine release, mice were given daily injection of 1 μg IL-25 during either early or more established atherosclerosis for seven days and killed one day later. The latter design was used to allow a better characterization of early immune responses to IL-25 treatment (Fig. 1).

### IL-25 treatment induces an expansion of ILC2s in the spleen

**Figure 2:**
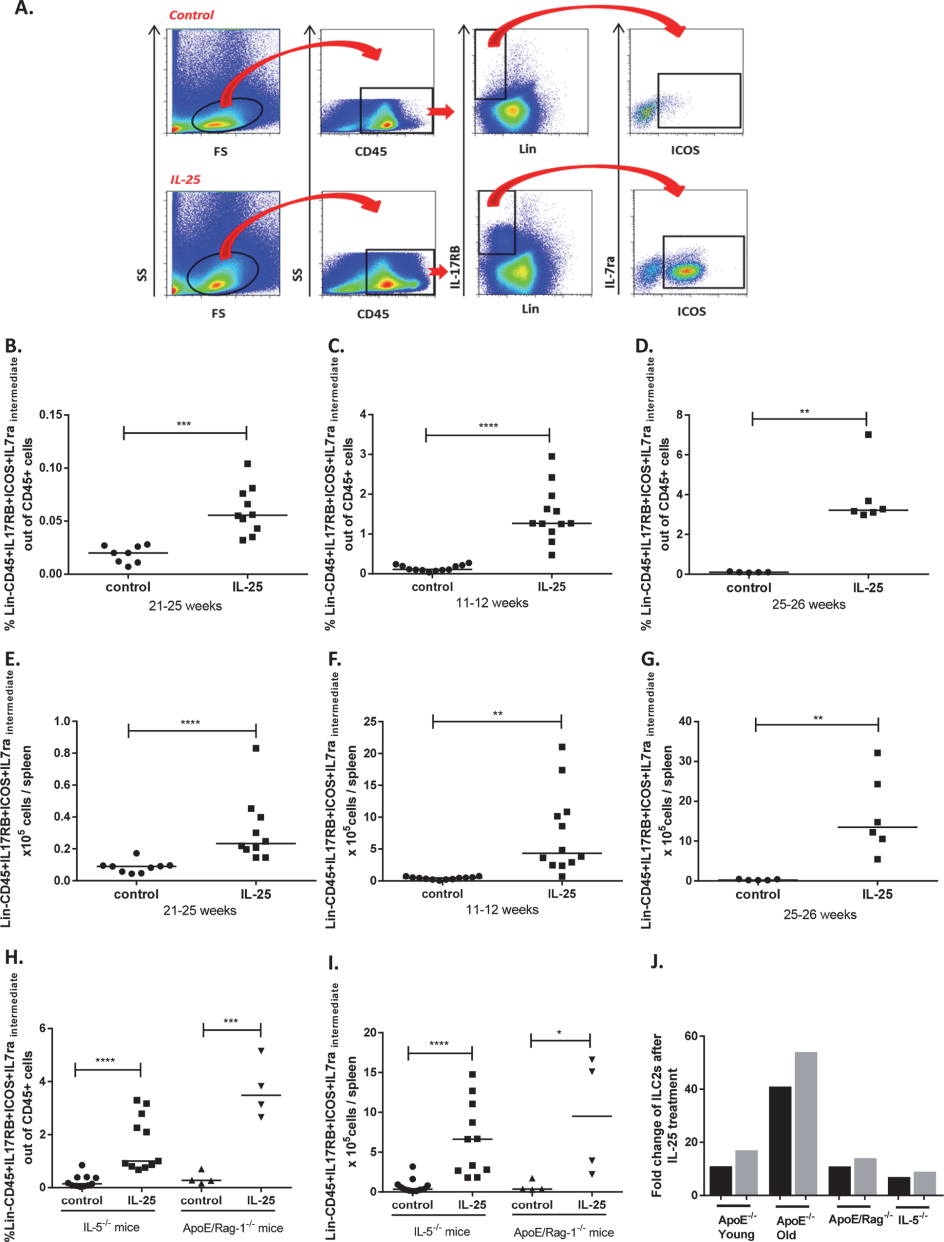
IL-25 induces an expansion of ILC2s in the spleen. **A**) Gating strategy to identify ILC2s in the spleen with flow cytometry. Leukocytes were gated in the forward (FS)/side scatter (SS) plot, and thereafter the CD45^+^, lineage negative cells (Lin-) expressing IL-17RB, intermediate IL-7ra and ICOS were identified. The ILC2s after a one-week treatment with 1μg rmIL-25 per day or equal volume of the control medium is shown. **B**) The frequency of ILC2s in the spleen after a 4-weeks treatment of old apoE deficient mice, **C**) the frequency of ILC2s in one-week treated young and **D**) old apoE deficient mice. **E**) The numbers of ILC2s in the spleen after a 4-weeks treatment of old apoE deficient mice, **F**) the numbers of ILC2s in one-week treated young and **G**) old apoE deficient mice. **H**) The frequency of ILC2s in the spleen of one-week treated apoE/Rag-1 deficient and IL-5 deficient mice. **I**) The numbers of ILC2s in the spleen of one-week treated apoE/Rag-1 deficient and IL-5 deficient mice. **J**) Fold change of the frequency (black bars) or numbers (grey bars) of ILC2s in the spleen of one-week IL-25 treated young (11–12 weeks of age) and old (25–26 weeks of age) apoE deficient, apoE/Rag-1 deficient and IL-5 deficient mice compared to the control mice, respectively. Each dot in the figure represents one mouse and the bar the median value. *P<0.05, **P<0.01; ***P<0.001, ****P<0.0001.

Recently it has been shown that IL-25 is a powerful inducer of the newly described innate cell population ILC2 [5]. Thus, we analyzed the ILC2 population in the spleen of IL-25 treated apoE deficient mice by flow cytometry (the gating strategy is shown in Fig. 2A). Interestingly, the ILC2 population was barely detected in control mice, whereas the IL-25 treatment resulted in a large increase of the ILC2s (Fig. 2B-G). Treatment with IL-25 for only one week was sufficient to induce a significant 11- and 41-fold increased frequency and 17- and 54-fold expansion in numbers of ILC2s in the spleen of young and old mice, respectively (Fig. 2J). This confirms that IL-25 induces expansion of ILC2s and shows that IL-25 is able to expand the ILC2 population also in non-hyperlipidimic as well as hyperlipidemic and atherosclerotic mice. To evaluate the role of the presence of T and B cells for the expansion of ILC2s, apoE/Rag-1 deficient mice were treated with IL-25. A one-week IL-25 treatment of apoE/Rag-1 deficient mice demonstrated an 11-fold increased frequency of the ILC2s in the spleen (Fig. 2H and 2J) as well as a 14-fold increase of ILC2 numbers (Fig. 2I-J).

### IL-25 treatment has limited influence on the T cell populations

Because IL-25 and ILC2s have been implicated in induction of Th2 immune responses the T cell populations of blood and spleens were examined to characterize the response to IL-25 treatment. To evaluate if IL-25 has any acute influence on Th1, Th2, Th17 and T regulatory cell populations, mice were given daily injections of 1μg IL-25 to both young and old apoE deficient mice for seven days and sacrificed one day later (Fig. 1). No differences in the Th1 (CD3^+^CD4^+^IFN-γ^+^), Th2 (CD3^+^CD4^+^IL-5^+^), Th17 (CD3^+^CD4^+^IL-17^+^) or the T regulatory (CD3^+^CD4^+^Foxp3^+^) cell populations were observed in either young or old mice treated with IL-25 for one week (S1 Table). However, splenocytes from one week IL-25 treated young mice were found to have reduced proliferative capacity (proliferation index of ConA stimulated and non-stimulated cells: 11±2 in IL-25 treated versus 18±7 in controls, *P* = 0.047). Also no differences in Th1 (14.3±3.2% versus 15.7±5.3% in IL-25 treated mice out of the CD3^+^CD4^+^ T cell population, *P* = 0.37), Th2 (3.4±1.9% versus 2.5±1.4% in IL-25 treated mice out of the CD3^+^CD4^+^ T cell population, *P* = 0.23) or Th17 cells (1.6±0.86% versus 2.1±0.9% in IL-25 treated mice out of the CD3^+^CD4^+^ T cell population, *P* = 0.14) were detected in the spleens of mice treated for 4-weeks (21–25 weeks of age) and sacrificed when 25-weeks old. On the other hand, an induction of the regulatory CD3^+^CD4^+^CD25^+^Foxp3^+^ T cell population in the spleen was detected in these old mice treated with IL-25 for 4-weeks (8.3±1.4% versus 10±2.5% in IL-25 treated mice out of the CD3^+^CD4^+^ T cell population, *P* = 0.04). No differences were detected in the Th1, Th2, Th17 and T regulatory cell populations of 4-week treated young mice in either blood or the spleen (data not shown). Taken together, this suggests that, apart from an increase in regulatory T cells in the spleen of old mice, IL-25 treatment has limited influence on the T cell populations in both blood and spleen.

### ILC2s produce IL-5 and IL-25 treatment increases IL-5 levels

**Figure 3:**
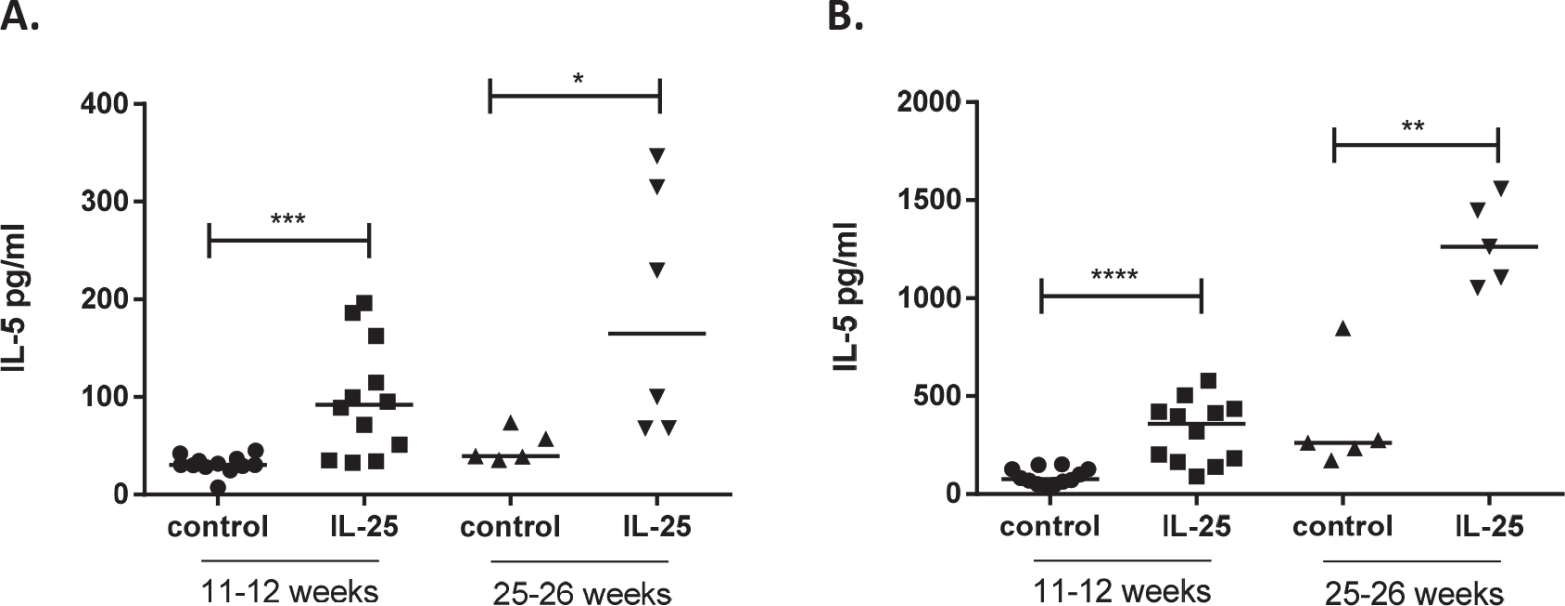
IL-25 increases IL-5 levels in blood and spleen. IL-5 levels in plasma and released from cultured splenocytes of young and old *Apoe* ^*-/-*^ mice treated with 1μg rmIL-25 per day or equal volume of the control medium during one week were analyzed by multiplex technology (Luminex). **A**) IL-5 in plasma and **B**) released from cultured splenocytes of IL-25 treated young and old mice. Each dot in the figure represents one mouse and the bar the median value. *P<0.05, **P<0.01, ***P<0.001. ****P<0.0001.

**Figure 4:**
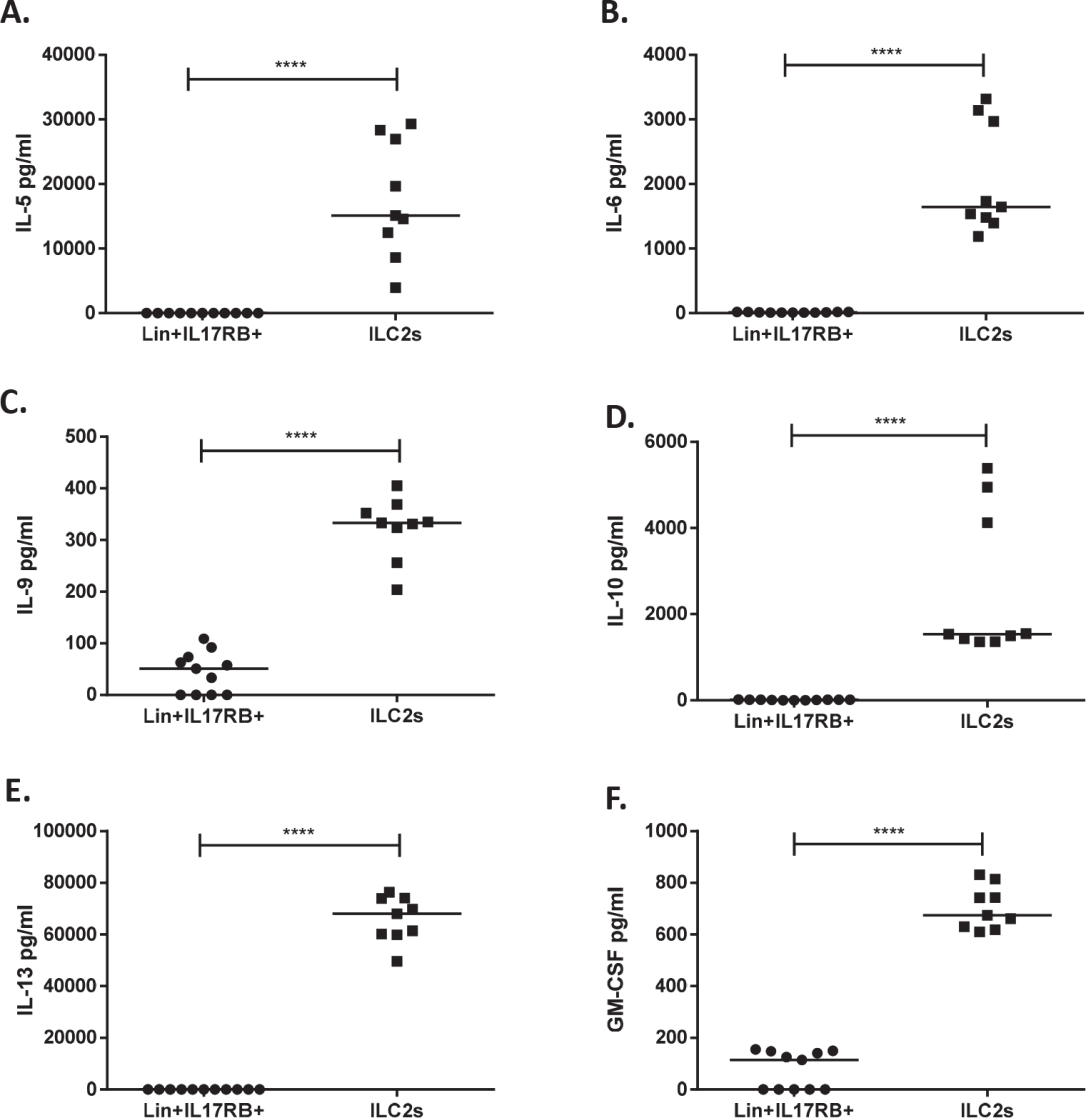
Cytokine release of FACS sorted ILC2s and Lin^+^CD45^+^IL17RB^+^ cells. ILC2s and Lin^+^CD45^+^IL17RB^+^ (Lin^+^IL17RB^+^) cells were sorted from the splenocytes of young *Apoe* ^*-/-*^ mice treated for one-week with 1 μg rmIL-25 per day. Upon sorting Lin^+^CD45^+^IL17RB^+^ cells (0.2 x 10^6^/well) were stimulated with PMA (20 ng/mL) and Ionomycin (1 μg/mL) for 24h. Sorted ILC2s were *in vitro* expanded in the presence of IL-7 (10 ng/mL) and IL-33 (10 ng/mL) for 7 days. Thereafter the *in vitro* expanded ILC2s (0.2 x 10^6^/well) were stimulated with PMA (20 ng/mL) and Ionomycin (1 μg/mL) for 24h. Release of **A**) IL-5, **B**) IL-6, **C**) IL-9, **D**) IL-10, **E**) IL-13 and **F**) GM-CSF was measured in the cell culture supernatants of stimulated ILC2s and Lin^+^CD45^+^IL17RB^+^ cells with the use of Luminex technology. Each dot in the figure represents one well of cells and the bar the median value. ****P<0.0001.

To examine if the presence of IL-25 has any impact on the systemic inflammatory response, cytokine levels were analyzed in blood and in supernatants of cultured splenocytes. ApoE deficient mice treated with daily injections of IL-25 for one week showed increased levels of IL-5 in blood compared to controls, both in young and old mice (Fig. 3A). In addition, upon a one-week treatment with IL-25, IL-12 plasma levels were reduced in old mice (215±27 pg/mL versus 135±31 pg/mL in IL-25 treated mice, *P* = 0.008), whereas IL-17 plasma levels were decreased in young mice (128±17 pg/mL versus 71±21 pg/mL in IL-25 treated mice, *P* = 0.004), suggesting that IL-25 promotes an anti-inflammatory cytokine response. No other differences in plasma cytokine levels were detected after the one-week treatments with recombinant IL-25 of apoE deficient mice (data not shown). Also increased plasma IL-5 levels were demonstrated in one-week IL-25 treated apoE/Rag-1 deficient mice (5.41±6.70 pg/mL versus 617.6±155.1 pg/mL in IL-25 treated mice, *P* = 0.03). In line with the findings in blood, cytokine levels in supernatants of cultured splenocytes from apoE deficient mice treated for one-week with IL-25 were found to contain higher levels of IL-5 (Fig. 3B). Release of other cytokines such as IFN-γ, IL-2, IL-4, IL-6, IL-9, IL-10, IL-12(p40), IL-13 and IL-17A was not affected (data not shown). The increased IL-5 levels support the notion that the IL-25 treatment induced an expansion of ILC2s in the spleen, since ILC2s responding to IL-25 have been found to be potent producers of IL-5 [6]. In further support, about 70% of sorted splenic ILC2s from one-week IL-25 treated young apoE deficient mice, cultured for 7 days in presence of IL-7 and IL-33 were found by flow cytometric analysis to express IL-5 and the ILC2s demonstrated release of extensive amounts of IL-5 compared to Lin^+^ CD45^+^IL17RB^+^ splenic cells (Fig. 4A). FACS-sorted and i*n vitro* expanded splenic ILC2s also released very high levels of IL-6, IL-9, IL-10, IL-13 and GM-CSF (Fig. 4B-F). These cytokines have previously been shown to be released from cultured ILC2s [8]. Additionally, isolated splenocytes, from both young and old apoE deficient mice, stimulated *in vitro* with IL-25 were found to release significantly higher levels of IL-5, IL-6 and IL-13 (S2A–C Fig.). Taken together, these findings suggest that IL-25 mainly induces ILC2-derived type 2 immune responses in atherosclerotic mice.

### IL-25 and ILC2s increase B1a cells in the spleen

**Figure 5:**
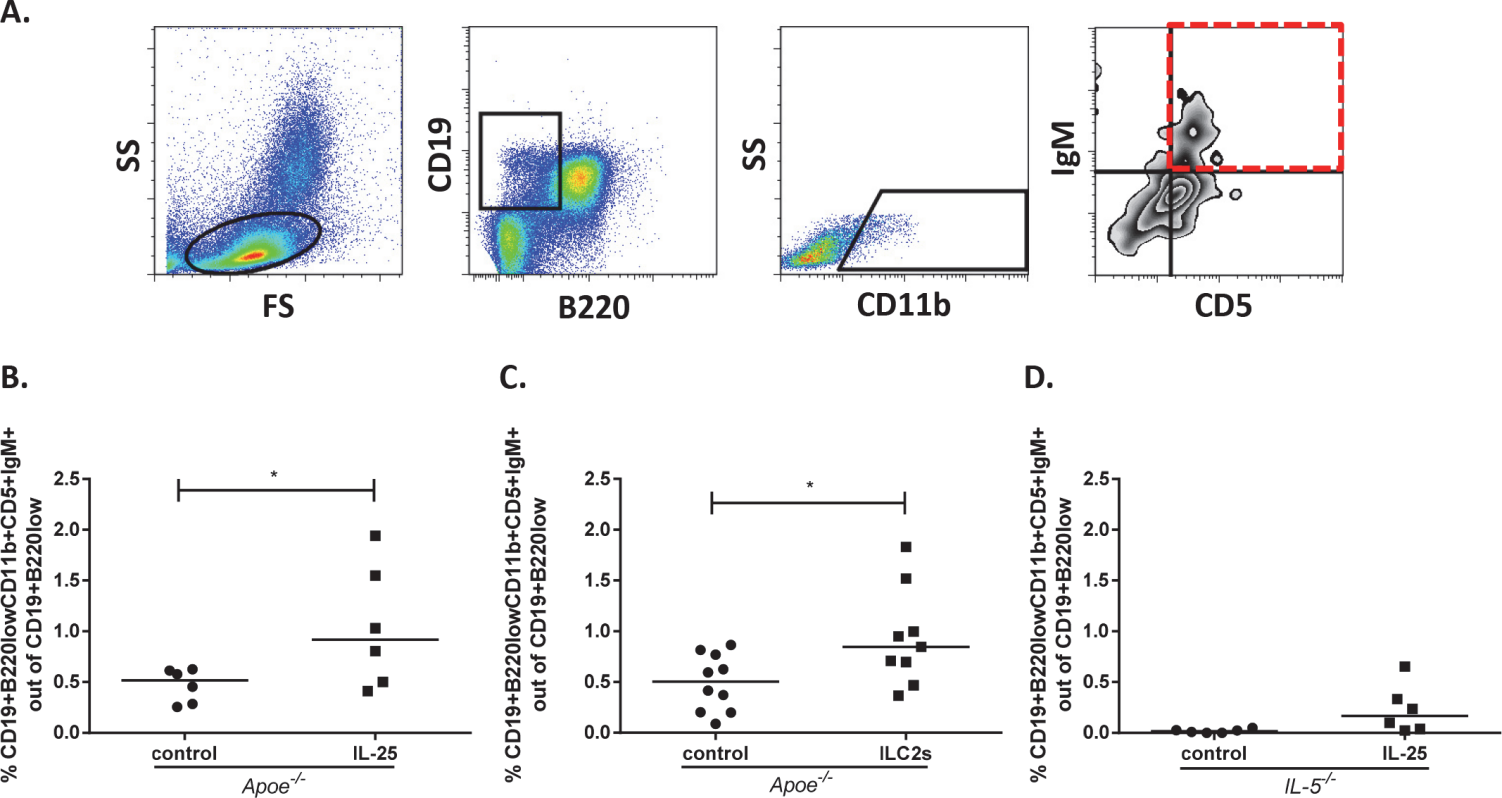
IL-25 and intra peritoneal transfer of ILC2s increase splenic B1a cells in *Apoe* ^*-/-*^ mice. **A**) Gating strategy to identify splenic B1a cells, gated as CD19^+^B220^low^CD11b^+^CD5^+^IgM^+^ with flow cytometry. **B**) Splenic B1a cells in *Apoe* ^*-/-*^ mice treated for one week (11–12 weeks of age) with 1μg rmIL-25 per day or equal volume of the control medium. **C**) FACS sorted ILC2s (Lin^-^CD45^+^IL17RB^+^ICOS^+^IL7ra^intermediate^) from the spleens of IL-25-treated *Apoe* ^*-/-*^ mice were expanded *in vitro* and transferred i.p. into young *Apoe* ^*-/-*^ mice (day 0). Controls were given the same volume of PBS. On day 3, mice were killed, spleens were dissected and analyzed by flow cytometry for B1a cells. **D**) Splenic B1a cells in *IL-5* ^*-/-*^ mice treated for one week (11–12 weeks of age) with 1μg rmIL-25 per day or equal volume of the control medium. Each dot in the figure represents one mouse and the bar the median value. *P<0.05.

It has previously been shown that IL-5 stimulates B1a cells to migrate to the spleen, where they differentiate into IgM-producing cells [12]. Thus, we were interested in studying if IL-25 and ILC2s influenced this cell population in the spleen (the gating strategy is shown in Fig. 5A). Old apoE deficient mice treated with IL-25 for 4-weeks (21–25 weeks of age) demonstrated an increase in splenic B1a cells (S3 Fig.), as well as young apoE deficient mice treated for one week (11–12 weeks of age) with IL-25 (Fig. 5B). Interestingly, intra-peritoneal transfer (day 0) of sorted and *in vitro* expanded ILC2s that were stained with Cell Trace Violet, were detected (day 3) in both spleen (S4 Fig.) and peritoneum (0.05% in PBS treated versus 0.15% Violet^+^ cells in ILC2 transferred mice) of apoE deficient mice. Additionally, transfer of sorted and i*n vitro* expanded ILC2s to young apoE deficient mice (11–12 weeks of age) was also found to increase the splenic B1a cell population, the main producer of natural IgM (Fig. 5C). On the other hand, only a small non-significant (*P* = 0.06) increase of splenic B1a cells was detected in IL-5 deficient mice, suggesting an important role of IL-5 for the induction of this cell population (Fig. 5D). However, a treatment with IL-5 alone had no direct effect on the levels of B1a cells in the spleen (0.72±0.55% B1a cells out of CD19^+^B220^low^ vs 0.56±31% in controls, *P* = 0.56).

### IL-25 increases plasma anti-PC IgM antibodies

**Figure 6:**
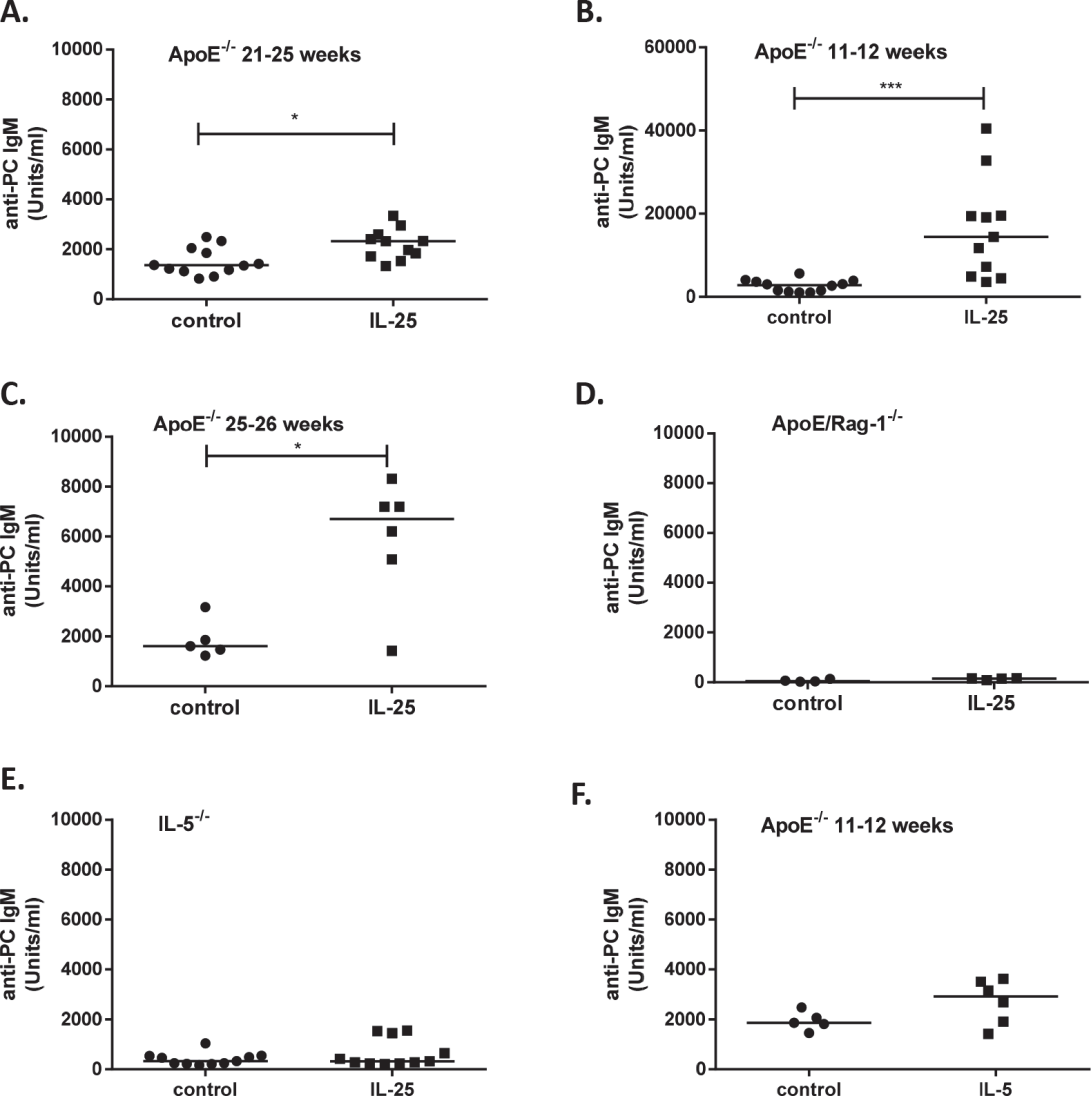
IL-25 increases plasma IgM anti-PC. IgM anti-phosphorycholine (PC) antibody levels in the plasma of mice treated with 1μg rmIL-25 per day or equal volume of the control medium for one or 4 weeks. **A**) 4-weeks IL-25 treatment of old *Apoe* ^*-/-*^ mice **B**) one-week IL-25 treatment of young *Apoe* ^*-/-*^ mice **C**) one-week IL-25 treatment of old *Apoe* ^*-/-*^ mice **D**) one-week IL-25 treatment of apoE/Rag-1 deficient mice **E**) one-week IL-25 treatment of IL-5 deficient mice and **F**) one-week IL-5 treatment of young *Apoe* ^*-/-*^ mice. Each dot in the figure represents one mouse and the bar the median value. *P<0.05, ***P<0.001.

PC is an important epitope on oxidized LDL and anti-PC IgM represents an extensively characterized natural IgM antibody. Interestingly, the 4-week-long IL-25 treatment of older apoE deficient mice with established disease showed increased levels of plasma anti-PC IgM (Fig. 6A) supporting our finding of elevated levels of B1a cells in the spleen. In addition, one-week-long treatments of apoE deficient mice also revealed elevated levels of these IgM antibodies (Fig. 6B-C). Taken together, these findings indicate that an athero-protective antibody response can be induced by IL-25. In contrast, the one-week long treatment of apoE/Rag-1 deficient mice lacking B and T cells these antibodies were barely detectable (Fig. 6D). Furthermore, in IL-5 deficient mice administration of IL-25 had no effect on the anti-PC IgM levels (Fig. 6E), indicating an important role of IL-5 for the production of these antibodies. However, treatment with IL-5 alone of young apoE deficient mice was not sufficient to induce significantly increased levels of anti-PC IgM (Fig. 6F). Notably, administration of IL-25 to IL-5 deficient mice expanded the frequency of ILC2s 7-fold in the spleen (Fig. 2H and 2J) and resulted in a 9-fold increase of ILC2 numbers (Fig. 2I-J), indicating that IL-5 is not required for the IL-25 induced expansion of ILC2s. Taken together, IL-25 administration induces anti-PC IgMs by an IL-5 dependent expansion of B1 cells that produce natural IgMs.

### IL-25 inhibits atherosclerosis development and stabilizes subvalvular plaques

**Figure 7:**
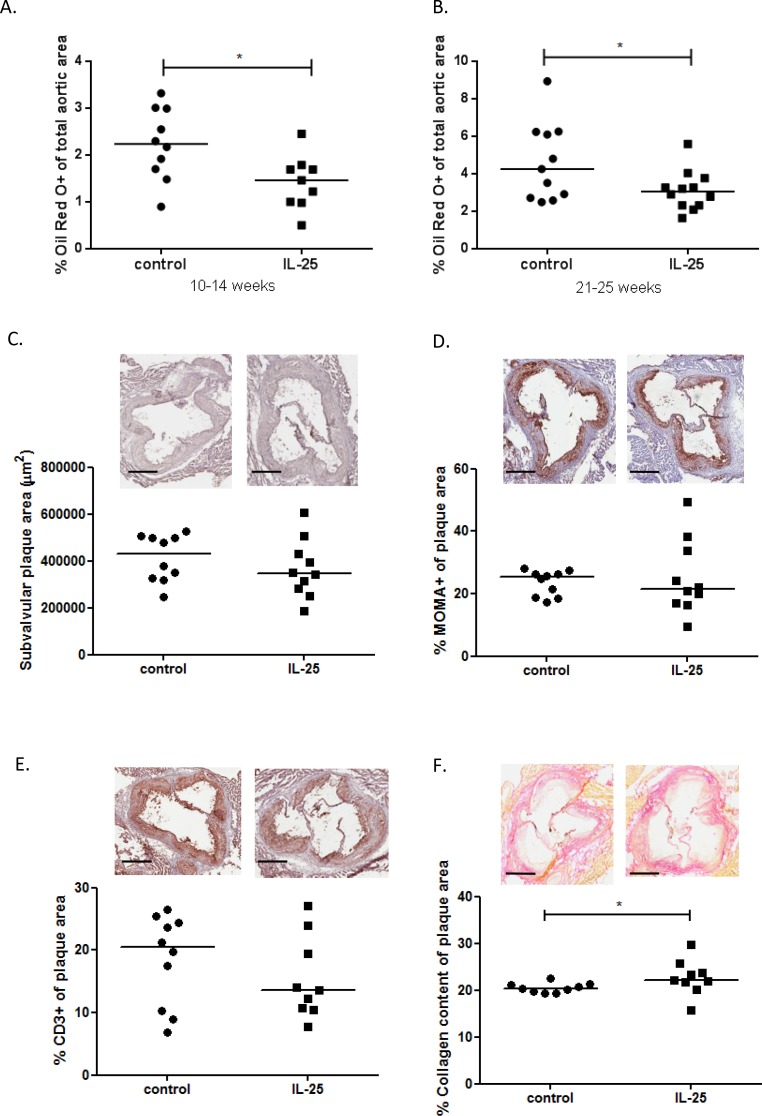
IL-25 treatment reduces atherosclerotic plaques in the aorta and stabilizes subvalvular plaques. Percentage of Oil red O positive plaque area staining in the whole aorta of *Apoe* ^*-/-*^ mice, fed a high fat diet from 10 weeks of age. The mice were treated daily with 1μg rmIL-25 or equal volume of the control medium for four weeks during (**A**) early atherosclerosis development or (**B**) during more established disease. Analysis of the subvalvular plaque composition in *Apoe* ^*-/-*^ mice treated with 1μg rmIL-25 per day or equal volume of the control medium for 4-weeks (10–14 weeks of age) and killed at the age of 25 weeks, **C**) Subvalvular plaque areas (μm^2^), **D**) percentage of macrophage positive plaque areas (MOMA), **E**) percentage of CD3^+^ T cell plaque areas and **F**) percentage of collagen positive plaque areas. Representative photomicrographs are included for each staining (Scale bar = 400 μm). All staining areas are calculated as percentage of total plaque area. Each dot in the figure represents one mouse and the bar the median value. *P<0.05.

In clinical studies, high levels of natural IgM anti-PC have been suggested to have a protective role against development of myocardial infarction [27]. Atherosclerosis-prone apoE deficient mice were administrated IL-25 during both early (10–14 weeks of age) and late (21–25 weeks of age) stages of atherosclerosis to evaluate if IL-25 have any influence on the plaque initiation or plaque progression (Fig. 1). IL-25 treatment significantly reduced initiation (Fig. 7A) and progression (Fig. 7B) of atherosclerosis development. The influence of IL-25 on plaque size was not dependent on cholesterol levels, since no differences between groups were detected (4-weeks treated young apoE deficient mice 735±82 vs 698±149 mg/dL in controls, *P* = 0.40; and 4-weeks treated old apoE deficient mice 553±116 vs 554±107 mg/dL in controls, *P* = 0.85). To evaluate if IL-25 influences plaque composition, inflammation or stability, we analysed macrophage, CD3^+^ T cell and collagen content in subvalvular lesions of 4-weeks treated (10–14 weeks of age) apoE deficient mice killed at 25 weeks of age. No differences were detected in subvalvular plaque size (Fig. 7C) or in infiltration of macrophages (Fig. 7D) or CD3^+^ T cells (Fig. 7E). Also no differences in the necrotic cores were detected (32.36±10.57% vs 36.76±11.53% acellular area of total plaque area in controls, *P* = 0.44). However, the collagen content (Fig. 7F) was slightly increased in plaques of IL-25 treated mice, suggesting an induction of increased lesion stability.

### IL-25 increase plasma IgA and IgE antibodies

IL-25 treatment of C57BL/6 mice has previously been shown to result in an increase of circulating IgA, IgE and IgG1 antibodies [1]. In the present study, we found increased levels of IgE in young apoE deficient mice treated with IL-25 for one week (11–12 weeks of age) compared to controls (174±124 versus 25±41 ng/ml in controls, *P* = 0.012). No differences were found in any of the other immunoglobulins tested (IgM, IgA, IgG1, IgG2b, IgG3; data not shown). In older apoE deficient mice with more established disease treated with IL-25 for 4-weeks (21–25 weeks of age), increased levels of IgA (48±16 versus 77±24 μg/ml in IL-25 treated mice, *P* = 0.001) and decreased levels of IgG2b (387±110 versus 276±71 μg/ml in IL-25 treated mice, *P* = 0.007) were detected, whereas no differences were observed for the other immunoglobulins (data not shown).

## Discussion

ILC2s are an emerging cell population with lymphoid morphology that can secrete cytokines that are also produced by Th2 cells, such as IL-5 and IL-13, and ILC2s are thought to represent an early source of these cytokines in type-2 immunity [5,6,8]. ILC2s have been implicated in wound healing and immunity to helminthes as well as allergic responses [5,6,8]. ILC2s are mainly targeted by the two type-2 immunity inducing cytokines, IL-25 and IL-33 [5]. In accordance, IL-25 induces production of the Th2 cytokines IL-4, IL-5 and IL-13 and functions as an initiator and promoter of Th2-cell mediated immune responses [1]. The purpose of this study was to evaluate if IL-25 has any influence on atherosclerosis development. Our findings show that IL-25 induces a large expansion of ILC2s in the spleen, increases splenic and plasma IL-5 levels as well as athero-protective anti-PC IgM in plasma and reduces atherosclerotic plaque development.

The present study demonstrates that IL-25 treatment resulted in extensive expansion of ILC2s in the spleen of both young and old apoE deficient mice. This was accompanied with increased levels of IL-5 in the circulation and the spleen. Thus, our data suggest that the ILC2s are the source of the increased IL-5 levels. Firstly, IL-25 treatment had limited influence on the T cell populations in both blood and spleen and, furthermore, we found that IL-25 treatment of apoE/Rag-1 deficient mice induced increased levels of ILC2s in the spleen and increased IL-5 levels in the plasma to the same extent as in T and B cell sufficient apoE deficient mice. Thus, it is unlikely that T and B cells are a source for the IL-25 induced increase in IL-5 levels. Secondly, the sorted and cultured splenic ILC2s of IL-25 treated apoE deficient mice released extensive amounts of IL-5 compared to Lin^+^CD45^+^IL17RB^+^ splenocytes. Thirdly, our findings demonstrate that splenocytes stimulated *in vitro* with IL-25 released IL-5, IL-6 and IL-13, a cytokine profile that previously has been reported from cultured ILC2s [8]. In accordance with our findings, it has recently been demonstrated that long-lived ILC2s in peripheral tissues are the predominant source of circulating IL-5 [28]. Together, the findings suggest that IL-25 treatment increases IL-5 levels as a result of the ILC2 expansion.

IL-25 has previously been shown to induce protection by the Th2 cytokines IL-4 and IL-13, in addition to IL-5 [29]. In our study, however, no differences in IL-13 levels were detected. The reason for this need to be further evaluated, but may reflect that the atherosclerotic environment influences the pattern of ILC2 released cytokines. The lack of IL-13 release in this study may also explain why no alterations in T helper subtype cells were observed.

IL-5 has been shown to stimulate the release of natural antibodies from B1 cells [25]. This is in line with our findings, that IL-25 treatment increased IL-5 and B1a cells in the spleen accompanied with elevated anti-PC IgM antibodies. In our study, administration of IL-25 to IL-5 deficient mice expanded ILC2s without a concomitant increase in natural anti-PC IgM, suggesting that ILC2 produced IL-5 is required for the production of these natural antibodies. Interestingly, the IL-5 treatment of apoE deficient mice was not as powerful as the IL-25 treatment in the induction of anti-PC IgM, suggesting a more potent role of IL-25 induced responses than IL-5 alone. Previous findings have shown that B1a cells stimulated with IL-5 migrate quickly to the spleen, where they differentiate into IgM-secreting cells [12]. We found that administration of IL-25 increased the number of B1a cells in the spleen. Furthermore, transfer of isolated ILC2s also resulted in higher levels of B1a cells in the spleen. Taken together, administration of IL-25 initiates a sequence of events involving expansion of ILC2s, increased IL-5 levels, B1a cell activation and a subsequent release of natural IgM.

Natural antibodies against the PC epitope have been shown to confer protection in experimental atherosclerosis in mice [30]. Anti-PC IgM has also been found to be associated with reduced cardiovascular risk in humans [27]. Also in a clinical study, plasma IL-5 levels were found to be related to levels of IgM antibodies recognizing oxidized LDL and to decreased subclinical atherosclerosis [31]. IL-25 has been shown to be expressed constitutively in normal human arteries and by resident plaque cells, suggesting that IL-25 may play a role in regulating local immune responses [32]. In our study, IL-25 treated mice were found to have a significantly reduced plaque area in the aorta, whereas no effect on atherosclerosis was detected in the more advanced subvalvular lesions. In line with the present findings several previous experimental studies have indicated variation of plaque development at different sites in apoE deficient mice [33]. Accordingly, the IL-25 treatment might have started too late in the atherosclerosis development to have any influence on the subvalvular plaques. In the analysis of the plaque content, no differences in presence of macrophages and T cells could be detected, but more collagen were found in the remaining plaques of the IL-25 treated mice, indicating increased plaque stability. In line with our findings, it has previously been demonstrated that airway smooth muscle cells activated with IL-25 increased their expression of extracellular matrix components [34].

ILC2s are also targeted by IL-33 [5]. Interestingly, IL-33 has been suggested to play a protective role in the development of atherosclerosis by inducing IL-5 and oxidized LDL specific antibodies [26]. In addition, blockade of OX40L reduced experimental atherosclerosis, induced IL-33 production by antigen presenting cells resulting in an increase of IL-5 and natural IgM specific for oxidized LDL [35]. A recent study demonstrated presence of B1a cells and natural helper cells in aortic samples from mice, and isolated aortic natural helper cells were found to produce IL-5 in response to IL-33 treatment [11]. This suggests that B1a cells could also be stimulated locally to produce athero-protective IgM natural antibodies.

IL-25 treatment of C57BL/6 mice has previously been shown to result in an increase of circulating IgA, IgE and IgG1 antibodies [1]. In line with these findings, we found increased levels of IgA and IgE in mice treated with IL-25. However, we found no differences in IgG1 plasma levels in contrast to the previous report. The reason for this discrepancy needs to be further evaluated, but may reflect an altered immune response taking place in our hypercholesterolemic mouse model, since the treatment did not induce Th2 immune responses. In addition, older mice with more established disease treated with IL-25 for 4 weeks also showed decreased levels of pro-inflammatory IgG2b antibodies.

One limitation of the present study is that the direct effect of ILC2s on atherosclerosis development cannot be analyzed due to the lack of mouse models specifically targeting the ILC2 cell population. ILC2s are present at low levels in Rag-deficient mice but can be expanded by IL-25 administration [8]. T and B cells have been suggested to have a role in the survival of ILC2s but not in their generation [8].

In the present study, we attribute IL-25 with a protective role in atherosclerosis. We show that administration of IL-25 induces an expansion of IL-5 releasing ILC2s in spleen resulting in increased levels of B1a cells and athero-protective IgM anti-PC antibodies rather than modulation of adaptive Th2 responses previously implicated in association with IL-25. Interestingly, high levels of IgM antibodies recognizing oxidized LDL have also been found to be associated with less atherosclerosis in humans. Therefore, if similar athero-protective immunity by IL-25 can be induced in humans, it would represent a possible novel therapeutic approach for prevention and treatment of cardiovascular disease.

## Supporting Information

S1 Table T cell subsets in blood and spleen of young and old *Apoe* ^-/-^ mice treated for one week with IL-25 or control medium. (DOCX)

S1 Fig Gating strategy to sort ILC2s and Lin^+^CD45^+^IL17RB^+^ cells with flow cytometry. From apoE deficient mice treated for one-week with 1μg rmIL-25 per day, lineage negative CD45^+^ cells in the spleen expressing IL-17RB, intermediate IL-7ra and ICOS were sorted. In detail, following enrichment of the splenic cells with the custom made kit from Stem cell Technologies in order to obtain lineage negative cells and a subsequent staining with the fluorochrome conjugated antibodies (Lin-Streptavidin PE/Cy7, ICOS-PB, CD45-APC/Cy7, IL-7ra-FITC, IL-17RB-APC), ILC2s were sorted with FACSAria (BD Biosciences) as Lin^-^CD45^+^IL17RB^+^ICOS^+^IL7ra ^intermediate^ together with a simultaneous sorting of Lin^+^CD45^+^IL17RB^+^ cells.
(TIF)

S2 Fig IL-25 induces release of IL-5, IL-6 and IL-13. Young and old *Apoe* ^*-/-*^ mice treated for one week with control medium were used for the isolation of splenocytes. The isolated splenocytes stimulated *in vitro* with IL-25 (50 ng/mL) were found to release significantly higher levels of **A**) IL-5, **B**) IL-6 and **C**) IL-13 compared to the vehicle stimulation (vehicle = 4 mmol/L HCl containing 0.1% BSA). Each dot in the figure represents one mouse and the bar the median value. *P<0.05, **P<0.01, ***P<0.001, ****P<0.0001.
(TIF)

S3 Fig IL-25 increases B1a cells in the spleen. Old *Apoe* ^*-/-*^ mice treated with 1μg rmIL-25 per day or equal volume of the control medium for 4 weeks demonstrated an IL-25 induced increase of B1a cells in the spleen gated as B220^low^CD19^+^CD5^+^IgD^-^. Each dot in the figure represents one mouse and the bar the median value. *P<0.05.
(TIF)

S4 Fig Transferred ILC2s migrate to the spleen. Flow cytometry plots of Cell Trace Violet^+^ cells in the spleen of *Apoe* ^*-/-*^ mice that received intra-peritoneal injections of either **A**) PBS or **B)** Cell Trace Violet^+^ ILC2s. Transfer of sorted and *in vitro* expanded ILC2s (0.5x10^6^) that were stained with Cell Trace Violet to *Apoe* ^*-/-*^ mice (day 0) were detected by the use of flow cytometric analysis in the spleen 3 days later.
(TIF)
