# Supplementary material for: IL-25 Inhibits Atherosclerosis Development in Apolipoprotein E Deficient Mice
Source: PLoS One. 2015 Jan 28;10(1):e0117255. doi: 10.1371/journal.pone.0117255 (PMC4309452; doi:10.1371/journal.pone.0117255)
Supplement: S1 Table — (DOCX) [file pone.0117255.s001.docx]

**Supporting Information Table**

**Table S1**. T cell subsets in blood and spleen of young and old *Apoe^-/-^* mice treated for one week with IL-25 or control medium.

**Young *Apoe^-/-^* mice**

| ***% out of CD3^+^CD4^+^*** | ***Control (mean±SD)*** | ***IL-25 (mean±SD)*** | ***P value*** |
| --- | --- | --- | --- |
| CD3^+^CD4^+^IFNγ^+^ (Th1, spleen) | 6.0 ±3.9 | 10.3 ±4.9 | 0.093 |
| CD3^+^CD4^+^IL5^+^ (Th2, spleen) | 11.7 ±5.1 | 13.7 ±7.3 | 0.788 |
| CD3^+^CD4^+^IL17^+^ (Th17, blood) | 2.2 ±0.88 | 4.2 ±3.9 | 0.423 |
| CD3^+^CD4^+^FoxP3^+^ (Tregs, blood) | 4.0 ±2.1 | 5.7 ±2.9 | 0.310 |

**Old *Apoe^-/-^* mice**

| ***% out of CD3+CD4+*** | ***Control (mean±SD)*** | ***IL-25 (mean±SD)*** | ***P value*** |
| --- | --- | --- | --- |
| CD3^+^CD4^+^IFNγ^+^ (Th1, spleen) | 16.7 ±5.2 | 14.7 ±4.0 | 0.398 |
| CD3^+^CD4^+^IL5^+^ (Th2, spleen) | 11.2 ±3.1 | 11.9 ±5.8 | 0.828 |
| CD3^+^CD4^+^IL17^+^ (Th17, blood) | 1.8 ±0.6 | 2.3 ±1.2 | 0.537 |
| CD3^+^CD4^+^FoxP3^+^ (Tregs, blood) | 7.8 ±5.0 | 4.2 ±2.3 | 0.247 |

SD, standard deviation
