# Supplementary figures and images for: IL-25 Inhibits Atherosclerosis Development in Apolipoprotein E Deficient Mice
Source: PLoS One. 2015 Jan 28;10(1):e0117255. doi: 10.1371/journal.pone.0117255 (PMC4309452; doi:10.1371/journal.pone.0117255)

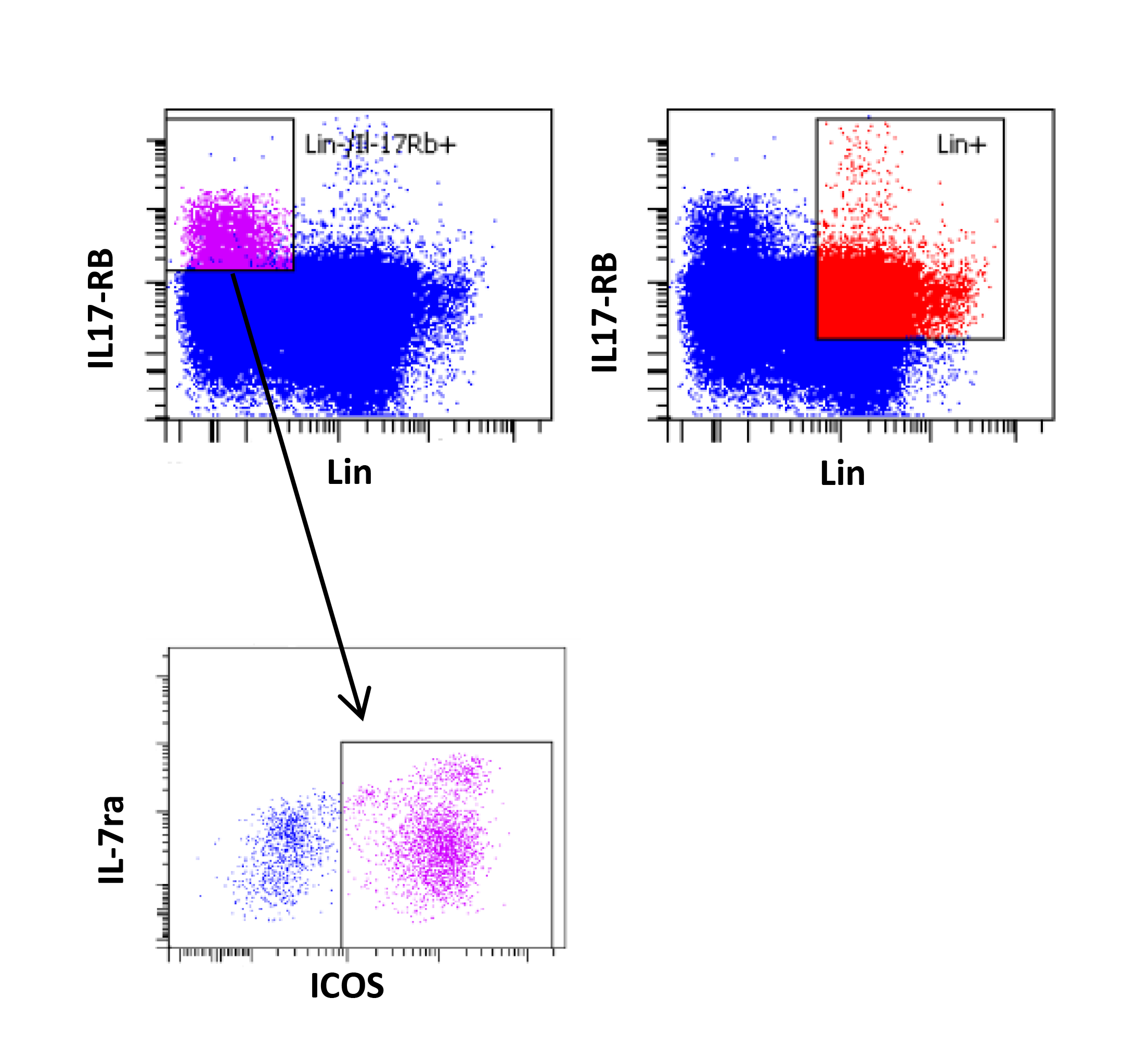

Supplement: S1 Fig — From apoE deficient mice treated for one-week with 1μg rmIL-25 per day, lineage negative CD45+ cells in the spleen expressing IL-17RB, intermediate IL-7ra and ICOS were sorted. In detail, following enrichment of the splenic cells with the custom made kit from Stem cell Technologies in order to obtain lineage negative cells and a subsequent staining with the fluorochrome conjugated antibodies (Lin-Streptavidin PE/Cy7, ICOS-PB, CD45-APC/Cy7, IL-7ra-FITC, IL-17RB-APC), ILC2s were sorted with FACSAria (BD Biosciences) as Lin-CD45+IL17RB+ICOS+IL7ra intermediate together with a simultaneous sorting of Lin+CD45+IL17RB+ cells. (TIF) [file pone.0117255.s002.tif]

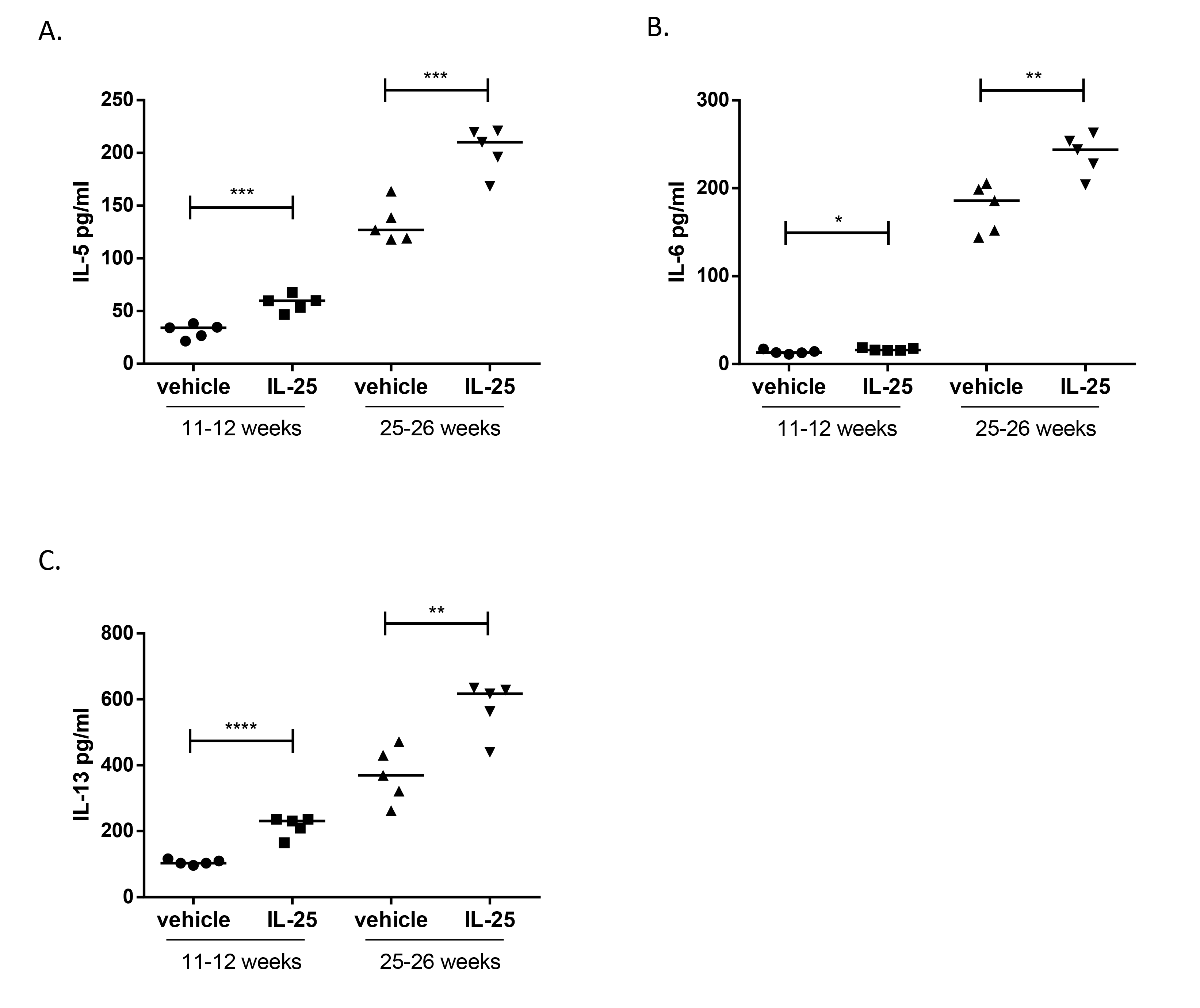

Supplement: S2 Fig — Young and old Apoe -/- mice treated for one week with control medium were used for the isolation of splenocytes. The isolated splenocytes stimulated in vitro with IL-25 (50 ng/mL) were found to release significantly higher levels of A) IL-5, B) IL-6 and C) IL-13 compared to the vehicle stimulation (vehicle = 4 mmol/L HCl containing 0.1% BSA). Each dot in the figure represents one mouse and the bar the median value. *P<0.05, **P<0.01, ***P<0.001, ****P<0.0001. (TIF) [file pone.0117255.s003.tif]

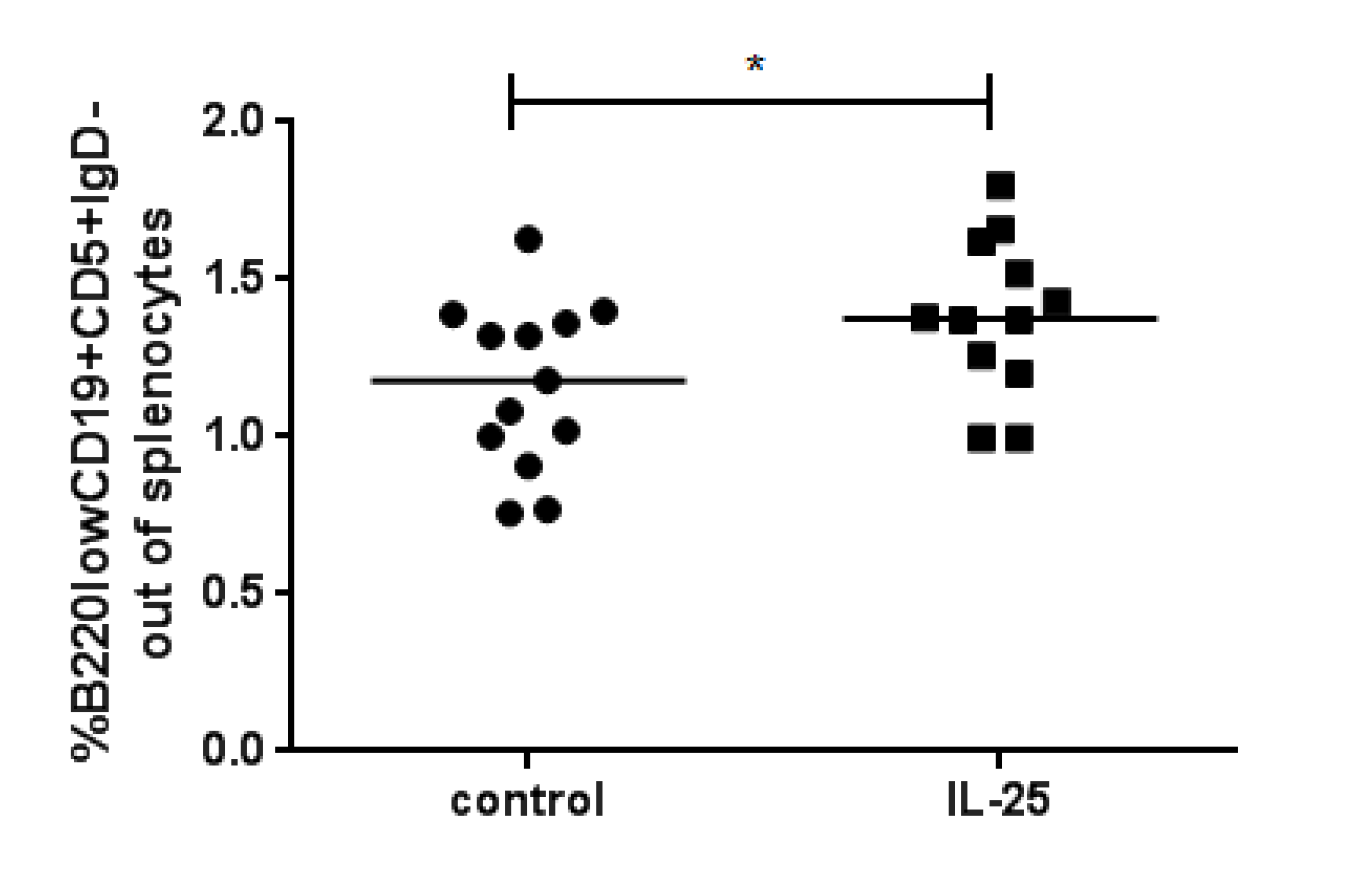

Supplement: S3 Fig — Old Apoe -/- mice treated with 1μg rmIL-25 per day or equal volume of the control medium for 4 weeks demonstrated an IL-25 induced increase of B1a cells in the spleen gated as B220lowCD19+CD5+IgD-. Each dot in the figure represents one mouse and the bar the median value. *P<0.05. (TIF) [file pone.0117255.s004.tif]

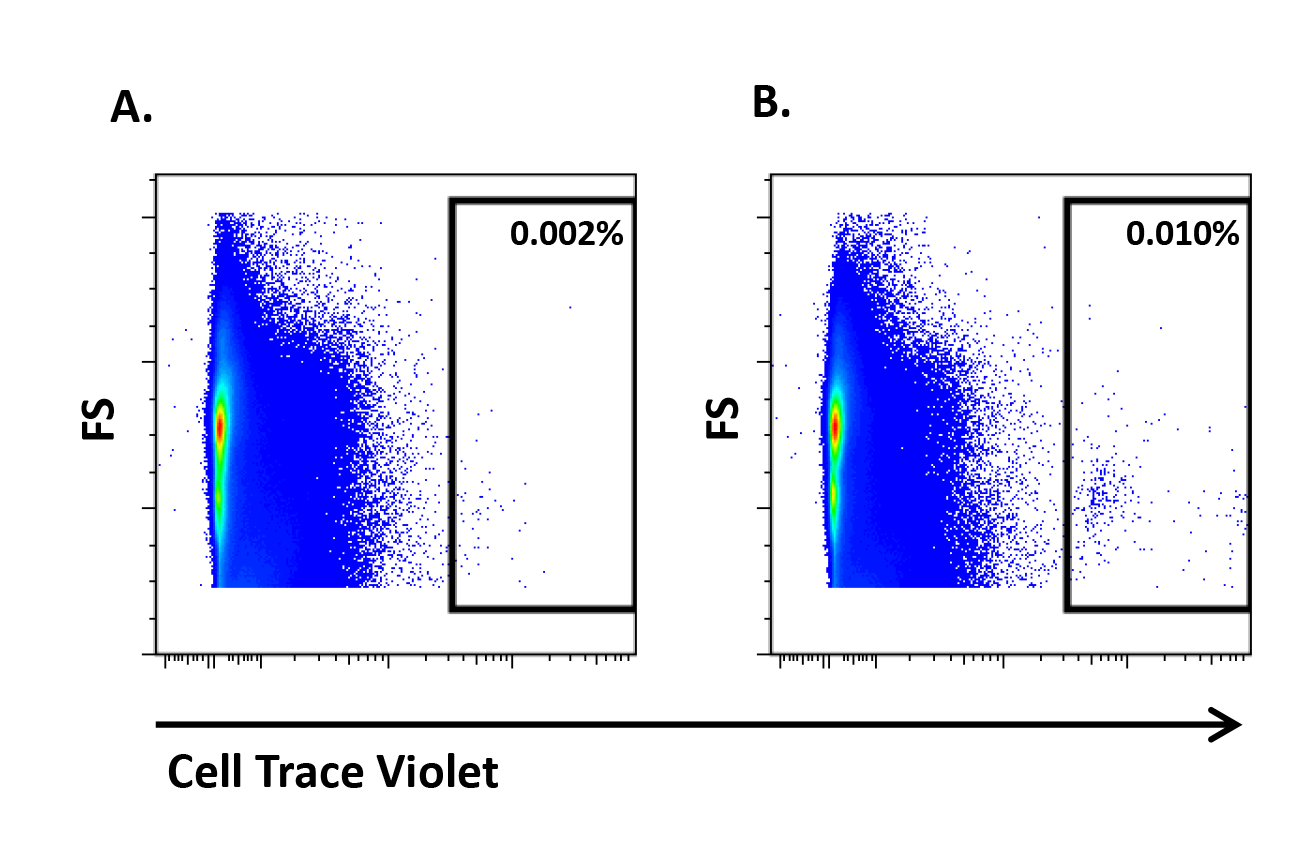

Supplement: S4 Fig — Flow cytometry plots of Cell Trace Violet+ cells in the spleen of Apoe -/- mice that received intra-peritoneal injections of either A) PBS or B) Cell Trace Violet+ ILC2s. Transfer of sorted and in vitro expanded ILC2s (0.5x10^6^) that were stained with Cell Trace Violet to Apoe -/- mice (day 0) were detected by the use of flow cytometric analysis in the spleen 3 days later. (TIF) [file pone.0117255.s005.tif]
